# Low Muscle and High Fat Percentages Are Associated with Low Natural Killer Cell Activity: A Cross-Sectional Study

**DOI:** 10.3390/ijms241512505

**Published:** 2023-08-06

**Authors:** A-Ra Cho, Eunkyung Suh, Hyoju Oh, Baek Hwan Cho, Minchan Gil, Yun-Kyong Lee

**Affiliations:** 1 Department of Family Medicine, Gangnam Severance Hospital, Yonsei University College of Medicine, Seoul 06273, Republic of Korea; ara1713@yuhs.ac; 2 Chaum Life Center, CHA University, Seoul 06062, Republic of Korea; sherby@chamc.co.kr (E.S.); a196019@chamc.co.kr (H.O.); 3 Department of Biomedical Informatics, CHA University School of Medicine, CHA University, Seongnam 13488, Republic of Korea; baekhwan.cho@cha.ac.kr; 4 NKMAX Co., Ltd., Seongnam 13605, Republic of Korea; minchangil@nkmax.com

**Keywords:** natural killer cell activity, obesity, body composition, fat percentage, muscle percentage, innate immunity

## Abstract

This study aimed to investigate whether body fat and muscle percentages are associated with natural killer cell activity (NKA). This was a cross-sectional study, conducted on 8058 subjects in a medical center in Korea. The association between the muscle and fat percentage tertiles and a low NKA, defined as an interferon-gamma level lower than 500 pg/mL, was assessed. In both men and women, the muscle mass and muscle percentage were significantly low in participants with a low NKA, whereas the fat percentage, white blood cell count, and C-reactive protein (CRP) level were significantly high in those with a low NKA. Compared with the lowest muscle percentage tertile as a reference, the fully adjusted odd ratios (ORs) (95% confidence intervals (CIs)) for a low NKA were significantly lower in T2 (OR: 0.69; 95% CI: 0.55–0.86) and T3 (OR: 0.74; 95% CI: 0.57–0.95) of men, and T3 (OR: 0.76; 95% CI: 0.59–0.99) of women. Compared with the lowest fat percentage tertile as a reference, the fully adjusted OR was significantly higher in T3 of men (OR: 1.31; 95% CI: 1.01–1.69). A high muscle percentage was significantly inversely associated with a low NKA in men and women, whereas a high fat percentage was significantly associated with a low NKA in men.

## 1. Introduction

Obesity is an excessive accumulation of body fat, which poses a risk for many diseases, including type 2 diabetes, cardiovascular disease [1], and at least 13 cancers [2], which have become serious health problems worldwide. In addition, it seems that patients with obesity are more susceptible to infections and infection-associated mortality [3,4]. Recent studies have reported that patients with obesity are more vulnerable to coronavirus disease 2019 infection, and are at a higher risk of severe complications and mortality [5]. Although the mechanisms underlying the link between obesity and infection are not fully established, an obesity-induced dysfunction in the innate and adaptive immune systems may be one of the hypotheses [4]. Obesity can cause the alteration and elevation of many adipokine productions, including leptin, adiponectin, tumor necrosis factor-α (TNF-α), and interleukin (IL)-6, within the adipose tissue, which can lead to chronic low-grade inflammation and, further, immune dysfunction [6,7].

Natural killer (NK) cells are a subset of innate lymphocytes, which play a role in protecting the host’s early immune function [8]. NK cells rapidly respond to infected or transformed cells by producing lytic molecules, such as perforins or granzymes, and can also cause immune responses through the rapid production of various cytokines, among which interferon-gamma (IFN-γ) is the best characterized [9]. A low level of NK cell activity is reported to be related to the development of infections, and also death, due to infection in immunologically normal subjects [10,11]. It has been seen in various tumors, with the association being more significant as the disease state is more advanced [12,13,14]. Several studies have investigated NK cell function in obesity. In vitro analyses have revealed an alteration in cytotoxicity and cytokine secretion, including that of IFN-γ, in human NK cells after incubation with adipokines such as leptin and adiponectin [15,16,17]. Several observational studies have shown impaired cell phenotypes, subset alterations, and cell functions in NK cells in individuals with obesity [18,19,20,21], whereas several other studies have reported no associations [22]. Moreover, clinical trial studies have demonstrated that the decrease in NK cell function has been significantly reversed in individuals with obesity after weight loss via bariatric surgery [23], or via dietary and exercise intervention [24]. A recent study has found a reduced production of IFN-γ by NK cells in obese patients, compared to the healthy control, and an increase in IFN-γ production after glucagon-like peptide-1 therapy [25].

Some limitations of these previous studies are small sample sizes, and the complexity of analyzing NK cell activity (NKA), which requires the isolation of peripheral blood mononuclear cells [26]. A relatively simple novel assay that uses whole blood has been recently developed for commercial use, to measure the NKA. This assay measures the amount of IFN-γ released by the activated NK cells contained in 1 mL of peripheral blood [27]. In addition, most of these previous studies included subjects with obesity defined using body mass index (BMI). BMI is considered to be the most widely used indicator of obesity. However, the same BMI can represent different body compositions, associated with different health outcomes, which is a major limitation of BMI as an obesity indicator [28,29,30]. Only one clinical study has shown a negative correlation between body fat percentage and NKA as part of the findings [31].

Therefore, this study aimed to investigate whether body fat and muscle percentages as body composition parameters are associated with NKA in a large population of healthy women and men, using a novel assay to measure the NKA.

## 2. Results

### 2.1. Clinical Characteristics of Study Population

Table 1 shows the clinical characteristics of the study population. In both men and women, the muscle mass and muscle percentage were significantly lower in subjects with a low NKA, whereas the fat percentage, white blood cell (WBC) count, and C-reactive protein (CRP) level were significantly higher in those with a low NKA. No significant differences in BMI were observed between subjects with a normal and low NKA, in both men and women. In men, the average age was significantly higher, and the average height and weight were significantly lower in subjects with a low NKA.

**Figure 1:**
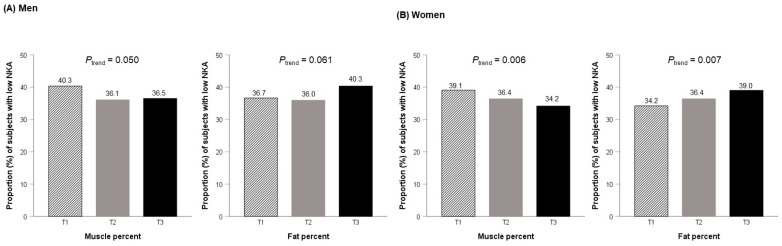
The proportion of subjects with a low NKA according to tertiles of muscle and fat percentages by gender. *p* for trend was estimated via the Cochran–Armitage test.

Figure 1 shows the proportion of subjects with a low NKA, according to the tertiles of muscle and fat percentages by gender. In men, the prevalence of a low NKA was 40.3%, 36.1%, and 36.5% in T1, T2, and T3 of muscle percentage, and 36.7%, 36.0%, and 40.3% in T1, T2, and T3 of fat percentage, respectively. In women, the prevalence of a low NKA was 39.1%, 36.4%, and 34.2% in T1, T2, and T3 of muscle percentage, and 34.2%, 36.4%, and 39.0% in T1, T2, and T3 of fat percentage, respectively. The proportion of low NKA decreased with increasing muscle percentage tertiles, and increased with increasing fat percentage tertiles only in women (*p* for a trend < 0.05 via the Cochran–Armitage test).

### 2.2. Correlations between IFN-γ Levels and Other Variables, Including Muscle and Fat Percentages

Table 2 shows the Pearson correlation coefficients between the IFN-γ levels and other clinical variables, including the muscle and fat percentages. The muscle mass (*r* = 0.079 in men; *r* = 0.032 in women) and muscle percentage (*r* = 0.049 in men; *r* = 0.053 in women) were positively correlated with the IFN-γ levels, whereas the fat percentage (*r* = −0.050 in men; *r* = −0.053 in women), WBC count (*r* = −0.169 in men; *r* = −0.225 in women), CRP level (*r* = −0.037 in men; *r* = −0.059 in women), insulin level (*r* = −0.048 in men; *r* = −0.046 in women), and homeostasis model assessment of insulin resistance (HOMA-IR) (*r* = −0.045 in men; *r* = −0.039 in women) were negatively correlated with the IFN-γ levels in both men and women. In men, the age was negatively correlated with the IFN-γ levels, whereas the height and weight were positively correlated with the IFN-γ levels.

### 2.3. Relationship between Tertiles of Muscle and Fat Percentages and Low NKA

Table 3 shows the multivariable logistic regression analysis results of the relationship between the muscle and fat percentage tertiles and a low NKA in men and women. Compared with the lowest muscle percentage tertile as a reference, the unadjusted ORs (95% CIs) for low NKA of T2 in men (OR: 0.84; 95% CI: 0.71–0.98) and T3 in women (OR: 0.81; 95% CI: 0.70–0.94) were significantly lower. After adjusting for age and BMI, the adjusted ORs (95% CIs) for low NKA were significantly lower in T2 (OR: 0.76; 95% CI: 0.63–0.91) and T3 (OR: 0.74; 95% CI: 0.61–0.92) of men, and T3 (OR: 0.76; 95% CI: 0.62–0.93) of women, compared with the reference. After additional adjustment for mediating factors, WBC count, and HOMA-IR, a similar trend was observed. The fully adjusted ORs (95% CIs) for low NKA were 0.69 (0.55–0.86) in T2, and 0.74 (0.57–0.95) in T3 of men, and 0.76 (0.59–0.99) in T3 of women. Compared with the lowest fat percentage tertile as a reference, the unadjusted OR (95% CI) for low NKA was significantly higher only in T3 of women (OR: 1.23; 95% CI: 1.06–1.43). After adjustment for age and BMI, the adjusted ORs for low NKA in T3 were significantly higher than those in T1 in both men (OR: 1.32; 95% CI: 1.08–1.63) and women (OR: 1.31; 95% CI: 1.07–1.61). However, after additional adjustment for WBC count and HOMA-IR, the fully adjusted OR was significantly higher only in T3 of men (OR: 1.31; 95% CI: 1.01–1.69).

## 3. Discussion

This study showed that an increase in muscle percentage was positively correlated with NKA, whereas an increase in fat percentage was negatively correlated with NKA, in both men and women. After adjustment for potential confounding variables, including inflammatory markers, a high muscle percentage was inversely associated with low NKA in both men and women, whereas a high fat percentage was associated with low NKA only in men. Previous studies have reported a lower NKA in obese subjects without any complications [18,21,32]. However, in this study, no significant differences in BMI were observed between participants with a normal and low NKA in both men and women, suggesting that body composition is more related to NKA than BMI is. Previous studies analyzing lean and adipose tissue compartments showed that the characterization of obesity based on BMI did not facilitate a comprehensive understanding of obesity-associated health risks [30]. Muller et al. showed high lean body mass as an important goal for disease prevention through body composition analysis, and suggested that the relationship between lean mass and health outcomes is due to immune function, pulmonary function, frailty, organ function, and thermoregulation [33].

The possible mechanisms of interaction between body composition and NKA are based on several effects of the adipose tissue and skeletal muscle on the immune system. The adipose tissue is an active endocrine organ secreting many adipokines, such as leptin, adiponectin, resistin, visfatin, TNF-α, and IL-6. The excessive increase in adipose tissue observed in obesity causes the dysregulation of adipokine secretion, which leads to chronic inflammation, and further contributes to the development of various diseases. It has been shown to affect the function of various immune cells, including NK cells [12,34,35]. Previous studies reported that NK cells showed a decreased secretion of the cytokines granzyme B and perforin, and an impaired degranulation capacity and cytotoxicity against malignant cells, in obese humans [21,32,34]. Recently, the skeletal muscle has been reported to play a role in regulating immune processes and inflammation responses [36]. Skeletal muscle cells modulate the immune system by signaling through various myokines, such as IL-6, IL-7, or IL-15, cell surface molecules, and cell-to-cell interactions [36,37]. Among these, IL-15 is important in the development and function of immune cells, and it seems to regulate the proliferation, activation, and distribution of NK cells [38].

Physical activity is one of the most effective interventions to reduce fat mass, increase body muscle mass, and decrease the risk of secondary diseases in individuals with obesity. Furthermore, regular moderate-intensity exercise has been reported to have immune-enhancing effects, through a reduction in inflammation, the maintenance of the thymic mass, alterations in immune cell compositions, enhanced immunosurveillance, and a reduction in psychological stress [39]. Many studies have shown an increased NK cell cytotoxicity during and after a moderate and intensive training program, including walking, running, marathons, bicycle racing, and aerobics [40,41,42,43,44]. Calorie restriction is another intervention for obesity prevention. The NK cell cytotoxicity has been reported to increase after energy restriction or a low-fat diet in individuals with obesity [45,46]. Additionally, the loss of body weight and body fat mass after bariatric surgery in patients with obesity has increased NK cell cytotoxicity and cytokine production [23].

This study showed that the fully adjusted OR was significantly lower in the highest tertile of muscle percentage in both men and women, whereas the fully adjusted OR was significantly higher in the highest tertile of fat percentage only in men. We adjusted for age, BMI, WBC count, and HOMA-IR as confounding variables. These variables were confirmed by the directed acyclic graph (DAG), a nonparametric graphical tool representing the causal relationship between exposure and outcomes. Although the precise mechanism has not been fully demonstrated, the differences according to sex might have contributed to our results. Previous studies have shown that chronic inflammation in obesity contributes to disease risk, which is significantly more robust in men, and women with obesity have more reduced proinflammatory adipose tissue macrophages than men with obesity [47]. Another study of men and women with obesity with metabolic syndrome showed that men had an excessive production of proinflammatory cytokines, whereas women had reduced levels of the anti-inflammatory adipokine adiponectin [48]. Furthermore, in this study, the mean value of BMI corresponded to obesity in men, and to normal weight in women, which could be a selection bias, as men with a higher BMI were included in this study.

To the best of our knowledge, there is no other study which has demonstrated the association between body fat and muscle percentage as body composition parameters, not simply BMI, and a low NKA. Moreover, the NKA was measured using a novel assay in a large population of healthy women and men. However, several inherent limitations are present. Firstly, while a relatively large sample sizes may be a major strength, this study was conducted from a single medical examination center, which may limit the generalizability of its results. Secondly, the evaluation of the NKA was performed only using an IFN-γ measurement in whole blood upon stimulation. While this new assay does not measure the cytotoxic capacity of the NK cells, like conventional techniques such as a ^51^Cr release assay and flow cytometry, it still has the advantages of simplicity and cost effectiveness, which can be more appropriate for large-scale studies. Thirdly, selection bias must be considered, due to the difference in the mean BMI between men and women. Fourthly, medical conditions that could affect the body composition, such as hypothyroidism or current use of weight loss medications, were not evaluated. Additionally, we could not assess clinical conditions, such as cigarette smoking, alcohol intake, physical activity, and recent use of nonsteroidal anti-inflammatory drugs or antibiotics, which may influence IFN-γ secretion. Lastly, the causal relationship could not be demonstrated, due to the cross-sectional study design. Thus, further longitudinal studies using both conventional techniques and the novel NKA assay are needed, to determine the clear causal relationship between body composition and NKA.

## 4. Materials and Methods

### 4.1. Study Population

This was a cross-sectional study analyzing data from Chaum Life Center. Individuals who underwent both an NKA assay and bioimpedance analysis at Chaum Life Center between January 2016 and May 2022 (n = 8154) were enrolled. Data were obtained from electronic medical records. From 8154 eligible subjects, 15 subjects under 19 years of age were excluded. Additionally, subjects with a history of malignant disease (n = 56), a history of autoimmune diseases such as rheumatoid arthritis or inflammatory bowel disease (n = 14), recent use of steroids or immunosuppressants (n = 5), or acute infectious disease (n = 6) were excluded. Finally, 8058 subjects (3682 men and 4376 women) were included in the analysis.

The study protocol was approved by the Institutional Review Board of CHA Bundang Medical Center (CHAMC 2020-10-006). This study was conducted in accordance with the principles of the Declaration of Helsinki.

### 4.2. Assessment of Body Composition

The body weight (to the nearest 0.1 kg) and height (to the nearest 0.1 cm) were measured while the subjects were wearing lightweight clothing and no shoes. The BMI was calculated as the ratio of weight (kg) to height squared (m^2^). The skeletal muscle and fat mass was measured using a bioimpedance analyzer (ACCUNIQ BC720, SELVAS Healthcare Inc., Daejeon, Republic of Korea), which was validated in previous studies as a reliable tool for the assessment of body composition [49,50]. The muscle and fat percentages were calculated by dividing the muscle and fat mass by the body weight, and multiplying by one hundred. The subjects were categorized into three groups, according to the sex-specific tertiles of muscle and fat percentages, as men and women showed significant differences in body composition (Online Supplementary Table S1). Muscle percentage: T1: <70.2%, T2: 70.2–74.6%, and T3; >74.6% in men, and T1: <63.4%, T2: 63.4–69.3%, and T3: >69.3% in women; fat percentage: T1: <21.1%, T2: 21.1–25.7%, and T3: >25.7% in men, and T1: <26.5%, T2: 26.5–32.6%, and T3: >32.6% in women.

### 4.3. IFN-γ Measurement for NKA

The NKA was evaluated through measurement of the IFN-γ released by activated NK cells, using a recently developed blood test (NK Vue^®^ Kit, NKMAX, Sungnam, Republic of Korea). A 1 mL sample of whole blood was transferred directly into a tube containing patented stimulatory cytokine (Promoca^®^, NKMAX, Sungnam, Republic of Korea), which specifically activates NK cells. Within 30 min of collection, the tube was gently and repeatedly mixed, and incubated for 20–24 h in a 37.0 °C chamber. During incubation, the stimulatory cytokine causes the secretion of IFN-γ into the plasma. Although only NK cells secrete a detectable amount of IFN-γ after Promoca^®^ stimulation [27], the interactions among activated immune cells might induce T cells and NKT cells to secrete IFN-γ. However, previous feasibility experiments have confirmed that IFN-γ secretion predominantly occurs through NK cells, rather than other innate or adaptive immune cells [27,51,52]. After incubation, the supernatant was obtained, centrifuged at 3000× *g* for 3 min, and then loaded onto enzyme-linked immunosorbent assay (ELISA) plates. The IFN-γ level was measured (pg/mL) using the designed ELISA. A low NKA was defined as an IFN-γ level lower than 500 pg/mL [53,54].

### 4.4. Data Collection

Blood samples were collected from the antecubital vein, in the morning, after at least 8 h of fasting. The WBC counts were quantified using a Sysmex XN-10 hematology analyzer (Sysmex, Lincolnshire, IL, USA). The CRP and fasting glucose levels were measured using a Hitachi 7600 analyzer (Hitachi Co., Tokyo, Japan). The fasting insulin level was measured via an electrochemiluminescence immunoassay, using an Elecsys 2010 instrument (Roche, Mannheim, Germany). The insulin resistance was estimated using the HOMA-IR method, based on the following formula [55]:

HOMA-IR = fasting insulin (μIU/mL) × fasting glucose (mg/dL)/405.

### 4.5. Statistical Analysis

The normality of the distribution of the variables was assessed using the Kolmogorov–Smirnov test. All data were presented as mean ± standard deviation. Clinical characteristics were compared between subjects with a normal and low NKA using *t*-tests. The proportion of low NKA was calculated according to the muscle and fat percentage tertiles in men and women, respectively. The *p* for a trend in the ordered tertile groups was evaluated using the Cochran–Armitage test. A simple Pearson correlation analysis was performed between the NKA and the other clinical variables, including the muscle and fat percentages.

**Figure 2:**
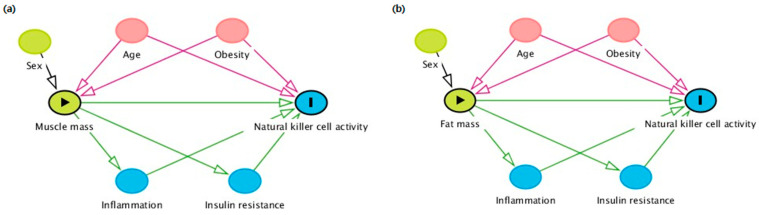
(**a**) Directed acyclic graph (DAG) of muscle mass and NKA, (**b**) DAG of fat mass and NKA. DAG representing the relationships among the exposure (muscle mass and fat mass; represented by the green oval with the triangle), outcome (NKA; represented by the blue oval with the line), and related factors. Variables represented as pink ovals are ancestors of exposure and outcome, variables represented as green ovals are ancestors of exposure, and variables represented as blue ovals are ancestors of outcome. Pink lines are biasing paths, and the green line between the exposure and outcome is the causal path of interest.

A theoretical causality model based on the DAG was constructed, considering the exposure variables (the muscle mass and fat mass), outcome variables (the NKA), possible confounders (age and obesity), and mediators (inflammation and insulin resistance), using the online software DAGitty version 3.2 (Figure 2a,b) [56]. Each variable in the DAG was chosen based on the literature review, and univariate analysis [21,53,57,58]. Multivariable logistic regression analysis was performed to calculate the odds ratios (ORs) and 95% confidence intervals (CIs) for low NKA in T2 and T3, compared to T1 as a reference, by gender. We adjusted for age and BMI in Model 1, and additionally adjusted for WBC count and HOMA-IR in Model 2. All the statistical analyses were performed using SPSS statistical software (version 25.0, SPSS Inc., Chicago, IL, USA). A *p*-value < 0.05 was considered statistically significant.

## 5. Conclusions

A high muscle percentage was independently and negatively associated with a low NKA in both men and women, whereas a high fat percentage was independently and positively associated with a low NKA only in men. These findings suggest that body composition parameters, such as body muscle and fat percentages modulate NK cell function. These results need to be interpreted with caution, but they may provide a valuable basis for further prospective studies to clarify the actual role of gender differences in NKA and body composition.

## Figures and Tables

**Table 1:** Clinical characteristics of the study population.

	Men			Women		
	Normal NKA	Low NKA	*p*	Normal NKA	Low NKA	*p*
Number, n	2296	1386		2777	1599	
Age, years	48.5 ± 11.5	50.2 ± 11.5	<0.001	47.1 ± 11.8	47.1 ± 11.6	0.960
Height, cm	174.2 ± 5.9	173.3 ± 5.9	<0.001	161.7 ± 5.7	161.5 ± 5.7	0.267
Weight, kg	76.5 ± 10.8	75.5 ± 11.8	0.007	56.7 ± 8.4	57.0 ± 9.6	0.351
BMI, kg/m^2^	25.2 ± 3.2	25.1 ± 3.5	0.288	21.7 ± 3.2	21.9 ± 3.6	0.206
Muscle mass, kg	55.0 ± 6.3	53.9 ± 6.6	<0.001	37.3 ± 4.1	37.0 ± 4.2	0.025
Muscle percent, %	72.3 ± 5.4	71.9 ± 5.7	0.026	66.4 ± 6.3	65.8 ± 6.5	0.001
Fat mass, kg	18.3 ± 6.5	18.5 ± 7.1	0.517	17.1 ± 6.1	17.7 ± 6.9	0.007
Fat percent, %	23.5 ± 5.7	24.0 ± 5.9	0.027	29.5 ± 6.6	30.2 ± 6.9	0.002
WBC, cells/μL	5.47 ± 1.38	5.99 ± 1.75	<0.001	4.99 ± 1.29	5.60 ± 1.63	<0.001
CRP, mg/dL	0.16 ± 0.32	0.21 ± 0.50	0.003	0.14 ± 0.28	0.21 ± 0.68	0.001
Glucose, mg/dL	94.7 ± 20.0	95.4 ± 21.0	0.329	85.6 ± 13.5	85.2 ± 15.1	0.404
Insulin, µIU/mL	7.5 ± 6.2	7.5 ± 5.4	0.973	5.3 ± 3.8	5.6 ± 4.3	0.091
HOMA-IR	1.84 ± 1.99	1.84 ± 1.70	0.967	1.18 ± 1.02	1.25 ± 1.12	0.109
IFN-γ, pg/mL	1665.4 ± 885.8	228.5 ± 140.2	<0.001	1692.9 ± 894.6	216.8 ± 142.6	<0.001
Hypertension, n (%)	500 (21.8)	295 (21.3)	0.424	233 (8.4)	137 (8.6)	0.545
Diabetes, n (%)	190 (8.3)	129 (9.3)	0.253	70 (2.5)	47 (2.9)	0.412
Dyslipidemia, n (%)	400 (17.4)	250 (18.0)	0.410	315 (11.3)	160 (10.0)	0.226

Data are expressed as mean ± SD. *p*-values were calculated using the *t*-test and chi-squared test. A low NKA is defined as an IFN-γ level lower than 500 pg/mL. Abbreviations: BMI, body mass index; CRP, C-reactive protein; HOMA-IR, homeostatic assessment model of insulin resistance; NKA, natural killer cell activity; WBC, white blood cell.

**Table 2:** Correlations between IFN-γ levels and other variables, including muscle and fat percentage, in each sex.

	Men		Women	
	*r*	*p*	*r*	*p*
Age, years	−0.068	<0.001	−0.020	0.178
Height, cm	0.050	0.002	0.025	0.102
Weight, kg	0.033	0.047	−0.019	0.208
BMI, kg/m^2^	0.014	0.409	−0.028	0.067
Muscle mass, kg	0.079	<0.001	0.032	0.033
Muscle percent, %	0.049	0.003	0.053	<0.001
Fat mass, kg	−0.026	0.116	−0.048	0.001
Fat percent, %	−0.050	0.002	−0.053	<0.001
WBC count, cells/μL	−0.169	<0.001	−0.225	<0.001
CRP, mg/dL	−0.037	0.047	−0.059	0.001
Glucose, mg/dL	−0.024	0.139	0.009	0.555
Insulin, µIU/mL	−0.048	0.018	−0.046	0.013
HOMA-IR	−0.045	0.024	−0.039	0.034

*r* is correlation coefficient, and *p*-values were calculated using Pearson’s correlation analysis. Abbreviations: BMI, body mass index; CRP, C-reactive protein; HOMA-IR, homeostatic assessment model of insulin resistance; WBC, white blood cell.

**Table 3:** Odd ratios and 95% confidence intervals of low NKA, according to the tertiles of muscle and fat percentage in each sex.

**Muscle Percent**	**T1**	**T2**		**T3**		
		**OR (95% CI)**	***p***	**OR (95% CI)**	***p***	**Overall *p***
Men						
Unadjusted	1 (Ref.)	0.84 (0.71–0.98)	0.030	0.85 (0.72–1.00)	0.051	0.056
Model 1	1 (Ref.)	0.76 (0.63–0.91)	0.002	0.74 (0.61–0.92)	0.005	0.005
Model 2	1 (Ref.)	0.69 (0.55–0.86)	0.001	0.74 (0.57–0.95)	0.019	0.005
Women						
Unadjusted	1 (Ref.)	0.89 (0.77–1.04)	0.136	0.81 (0.70–0.94)	0.006	0.022
Model 1	1 (Ref.)	0.86 (0.72–1.02)	0.090	0.76 (0.62–0.93)	0.009	0.033
Model 2	1 (Ref.)	0.93 (0.74–1.15)	0.491	0.76 (0.59–0.99)	0.042	0.087
**Fat Percent**	**T1**	**T2**		**T3**		
		**OR (95% CI)**	***p***	**OR (95% CI)**	***p***	**Overall *p***
Men						
Unadjusted	1 (Ref.)	0.97 (0.82–1.14)	0.715	1.17 (0.99–1.37)	0.063	0.056
Model 1	1 (Ref.)	1.00 (0.84–1.19)	0.989	1.32 (1.08–1.63)	0.008	0.005
Model 2	1 (Ref.)	0.92 (0.74–1.14)	0.439	1.31 (1.01–1.69)	0.043	0.009
Women						
Unadjusted	1 (Ref.)	1.10 (0.95–1.28)	0.215	1.23 (1.06–1.43)	0.007	0.026
Model 1	1 (Ref.)	1.13 (0.96–1.32)	0.141	1.31 (1.07–1.61)	0.010	0.038
Model 2	1 (Ref.)	1.21 (0.99–1.48)	0.061	1.29 (1.00–1.68)	0.054	0.100

Model 1: Adjusted for age and BMI. Model 2: Adjusted for age, BMI, WBC, and HOMA-IR. A low NKA is defined as an IFN-γ level lower than 500 pg/mL. Abbreviations: BMI, body mass index; HOMA-IR, homeostatic assessment model of insulin resistance; WBC, white blood cell.

## Data Availability

All datasets used and/or analyzed during the study are available from the corresponding author upon reasonable request.

## Supplementary Materials

The supporting information can be downloaded at: https://www.mdpi.com/article/10.3390/ijms241512505/s1.
