# Supplementary material for: Low Muscle and High Fat Percentages Are Associated with Low Natural Killer Cell Activity: A Cross-Sectional Study
Source: Int J Mol Sci. 2023 Aug 6;24(15):12505. doi: 10.3390/ijms241512505 (PMC10419953; doi:10.3390/ijms241512505)
Supplement: Supplementary file 1 [file ijms-24-12505-s001.zip › ijms-2520590-supplementary.pdf]

Table S1. Differences in body composition and clinical variables according to the gender.

	**Men**	**Women**	***p***
Number, n	3,682	4,376	
Age, years	49.2 ± 11.5	47.1 ± 11.7	<0.001
Height, cm	173.8 ± 5.9	161.6 ± 5.7	<0.001
Weight, kg	76.1 ± 11.2	56.8 ± 8.8	<0.001
BMI, kg/m^2	25.2 ± 3.3	21.8 ± 3.4	<0.001
Muscle mass, kg	54.6 ± 6.4	37.2 ± 4.1	<0.001
Muscle percent, %	72.2 ± 5.5	66.2 ± 6.4	<0.001
Fat mass, kg	18.4 ± 6.7	17.3 ± 6.4	<0.001
Fat percent, %	23.7 ± 5.8	29.8 ± 6.7	<0.001
WBC, cells/µL	5.66 ± 1.55	5.21 ± 1.45	<0.001
CRP, mg/dL	0.18 ± 0.40	0.16 ± 0.47	0.139
Glucose, mg/dL	94.9 ± 20.4	85.4 ± 14.1	<0.001
Insulin, µIU/mL	7.5 ± 5.9	5.4 ± 4.0	<0.001
HOMA-IR	1.84 ± 1.88	1.21 ± 1.06	<0.001
NKA, pg/mL	1124.5 ± 990.6	1153.5 ± 1010.3	0.195
Hypertension, n (%)	797 (21.6)	371 (8.5)	<0.001
Diabetes, n (%)	319 (8.7)	117 (2.7)	<0.001
Dyslipidemia, n (%)	650 (17.7)	475 (10.9)	<0.001

Data are expressed as mean ± SD. *p*-values were calculated using the t-test and chi-squared test.

Abbreviations: BMI, body mass index; CRP, C-reactive protein; HOMA-IR, homeostatic assessment model of insulin resistance; NKA, natural killer cell activity; WBC, white blood cell.
